# Gut Microbiota Contributes to Resistance Against Pneumococcal Pneumonia in Immunodeficient Rag^−/−^ Mice

**DOI:** 10.3389/fcimb.2018.00118

**Published:** 2018-04-18

**Authors:** Krysta M. Felix, Ivan A. Jaimez, Thuy-Vi V. Nguyen, Heqing Ma, Walid A. Raslan, Christina N. Klinger, Kristian P. Doyle, Hsin-Jung J. Wu

**Affiliations:** ^1^Department of Immunobiology, University of Arizona, Tucson, AZ, United States; ^2^Department of Neurology, College of Medicine, University of Arizona, Tucson, AZ, United States; ^3^Arizona Arthritis Center, College of Medicine, University of Arizona, Tucson, AZ, United States

**Keywords:** microbiota, *Streptococcus pneumoniae*, immunocompromised, gut-lung axis, segmented filamentous bacteria (SFB), neutrophils, CD47

## Abstract

*Streptococcus pneumoniae* causes infection-related mortality worldwide. Immunocompromised individuals, including young children, the elderly, and those with immunodeficiency, are especially vulnerable, yet little is known regarding *S. pneumoniae-*related pathogenesis and protection in immunocompromised hosts. Recently, strong interest has emerged in the gut microbiota's impact on lung diseases, or the “gut-lung axis.” However, the mechanisms of gut microbiota protection against gut-distal lung diseases like pneumonia remain unclear. We investigated the role of the gut commensal, segmented filamentous bacteria (SFB), against pneumococcal pneumonia in immunocompetent and immunocompromised mouse models. For the latter, we chose the Rag^−/−^ model, with adaptive immune deficiency. Immunocompetent adaptive protection against *S. pneumoniae* infection is based on antibodies against pneumococcal capsular polysaccharides, prototypical T cell independent-II (TI-II) antigens. Although SFB colonization enhanced TI-II antibodies in C57BL/6 mice, our data suggest that SFB did not further protect these immunocompetent animals. Indeed, basal B cell activity in hosts without SFB is sufficient for essential protection against *S. pneumoniae*. However, in immunocompromised Rag^−/−^ mice, we demonstrate a gut-lung axis of communication, as SFB influenced lung protection by regulating innate immunity. Neutrophil resolution is crucial to recovery, since an unchecked neutrophil response causes severe tissue damage. We found no early neutrophil recruitment differences between hosts with or without SFB; however, we observed a significant drop in lung neutrophils in the resolution phase of *S. pneumoniae* infection, which corresponded with lower CD47 expression, a molecule that inhibits phagocytosis of apoptotic cells, in SFB-colonized Rag^−/−^ mice. SFB promoted a shift in lung neutrophil phenotype from inflammatory neutrophils expressing high levels of CD18 and low levels of CD62L, to pro-resolution neutrophils with low CD18 and high CD62L. Blocking CD47 in SFB(−) mice increased pro-resolution neutrophils, suggesting CD47 down-regulation may be one neutrophil-modulating mechanism SFB utilizes. The SFB-induced lung neutrophil phenotype remained similar with heat-inactivated *S. pneumoniae* treatment, indicating these SFB-induced changes in neutrophil phenotype during the resolution phase are not simply secondary to better bacterial clearance in SFB(+) than SFB(−) mice. Together, these data demonstrate that the gut commensal SFB may provide much-needed protection in immunocompromised hosts in part by promoting neutrophil resolution post lung infection.

## Introduction

Pneumonia is the most common infectious cause of death worldwide, with almost 3.5 million deaths yearly (Wunderink and Waterer, 2014). *Streptococcus pneumoniae*, a species of Gram-positive bacteria that colonizes the upper respiratory tract, is the most common cause of pneumonia. One of the biggest concerns associated with *S. pneumoniae* infection is its high mortality in immunocompromised patients such as elderly, neonatal, and immunosuppressed patients (Lynch and Zhanel, 2010). Two types of vaccines against *S. pneumoniae* are available and these vaccines' efficacy relies heavily on their ability to induce an effective antibody response. However, the dilemma of controlling *S. pneumoniae* infections is that many individuals in the high risk populations that receive vaccination, such as neonates or the elderly, are also the populations that have intrinsic B cell defects and cannot mount a proper humoral response (Vinuesa et al., 2001; Aberle et al., 2013). Therefore, there is an urgent need to understand the pathogenesis of *S. pneumoniae* infection in immunocompromised hosts and alternative immune mechanisms that can provide protection in immunocompromised hosts. Recently, a strong interest has emerged in determining the impact of the gut microbiota in lung diseases. This has been referred to as the “gut-lung axis,” and is exemplified by the gut microbiota's influence on the development of lung diseases including respiratory infections, asthma, and allergies (Fujimura and Lynch, 2015; Budden et al., 2017). However, the cellular and molecular mechanisms by which the gut microbiota protects against gut-distal lung diseases such as pneumonia remain unclear.

One of the major immune cell types involved in *S. pneumoniae* infection is the neutrophil (De Filippo et al., 2014). During inflammation or infection, there is a shift toward “emergency hematopoiesis,” which increases the output of monocytes and neutrophils from the bone marrow or systemic lymphoid tissues such as the spleen (Bronte and Pittet, 2013; Boettcher and Manz, 2017), expanding the pool of circulating neutrophils that can be recruited to inflammatory sites for protection. However, what is often neglected is that for successful host immune protection to occur, neutrophil resolution at the end of the infection is just as important as neutrophil recruitment in the early phase of infection. Prolonged or excessive recruitment can be seriously detrimental to the host (Bhowmick et al., 2013; Bou Ghanem et al., 2015; Porto and Stein, 2016). Strict regulation of neutrophil pulmonary inflammation is essential; depletion of neutrophils at the beginning of an *S. pneumoniae* infection decreased host survival, while neutrophil depletion 18 h post infection significantly improved survival (Bou Ghanem et al., 2015). During the resolution phase of an inflammatory response, neutrophils undergo programmed apoptosis, and must be cleared from the tissues. One molecule that controls apoptotic neutrophil clearance by phagocytes, or “efferocytosis,” is CD47, a ubiquitously expressed “don't eat me” marker that interacts with signal regulatory protein α (SIRPα), a glycoprotein expressed on phagocytes, to inhibit efferocytosis (Barclay and Van den Berg, 2014).

The gut microbiota is essential in neutrophil development and homeostasis. Germ-free mice have a reduced neutrophil population in the blood compared to those colonized with even a few species of bacteria (Balmer et al., 2014). During infection, a pioneer study by Clarke et al. reported that microbiota-derived peptidoglycan systemically primes bone marrow-derived neutrophils, which enhances their killing of *S. pneumoniae* and *Staphylococcus aureus* (Clarke et al., 2010). Importantly, a single species of gut commensal, segmented filamentous bacteria (SFB), has been demonstrated to be sufficient to increase host resistance to *S. aureus* pneumonia by recruiting neutrophils in a Th17-dependent manner (Gauguet et al., 2015). Despite several reports of the microbiota enhancing host immune responses by priming and activating neutrophils, it is still unclear whether the gut microbiota participates in protecting the host from lung infection by controlling neutrophil resolution at the end stage of infection. Moreover, humoral immunity to T cell-independent type II (TI-II) antigens is critical for protection against encapsulated bacteria such as *S. pneumoniae*. Our lab has recently demonstrated that the gut commensal SFB enhances antibody production by inducing T follicular helper (Tfh) cells, a T cell subtype specialized in helping B cells, in autoimmune settings (Teng et al., 2016). Additionally, it has been reported that SFB induces germinal center formation and promotes gut IgA secretions (Thurnheer et al., 2003). However, little is known about whether there is a direct role for SFB in mediating a rapid and T cell-independent B cell response such as TI-II antibody production.

Here, we developed an animal model that examined the pathogenesis of and immune response against *S. pneumoniae*, with a focus on immunocompromised hosts. As *S. pneumoniae* infection occurs acutely, we hypothesized and tested whether the gut microbiota enhances host defense against *S. pneumoniae* infection by modulating innate or innate-like, rapid immune responses such as neutrophil and TI-II antibody responses. Though we found that SFB colonization enhanced TI-II antibody responses in wild type (WT) C57BL/6 (B6) mice, our data suggest that SFB did not provide further protection in these immunocompetent animals. However, in immunocompromised animals with adaptive immune deficiency, we demonstrate a gut-lung axis of communication, as SFB was able to provide lung protection through regulating innate immunity. We traced this protective effect to an efficient clearance of the lung neutrophil population at the resolution stage of the *S. pneumoniae* infection, which corresponds with lower expression of the anti-phagocytic molecule CD47, suggesting greater neutrophil resolution in SFB-colonized immunocompromised mice. Thus, these data demonstrate a novel protective effect of gut commensals: promoting neutrophil resolution and restoring them to basal levels after infection.

## Materials and methods

### Bacterial strains

*Streptococcus pneumoniae* ATCC 6303 (a type 3 strain) was obtained from the American Type Culture Collection (ATCC). For *in vivo* challenges, frozen bacterial stocks of *S. pneumoniae* were streaked out on Trypticase Soy Agar II (TSA-II, BD Biosciences) plates containing 5% sheep blood. A single colony was inoculated into 5 ml of Todd-Hewitt broth with 0.5% added yeast extract (THY; both from Sigma Aldrich), and grown at 37°C with 5% CO_2_ without shaking until bacteria reached an OD of 0.45–0.55 at 600 nm, representing ~10^8^ CFU/ml. Bacteria were spun down and resuspended in Dulbecco's Phosphate Buffered Saline (DPBS, Fisher Scientific), then diluted to the appropriate intended inoculum (7 × 10^4^-1.5 × 10^6^ CFU per mouse). 20 μl of bacterial suspension was inoculated per nare (40 μl total per mouse). The inoculum was verified after serial dilution in DPBS and plating on TSA-II plates with 5% sheep blood, incubated overnight at 37°C with 5% CO_2_.

### Mice

Tcrα^−/−^ and Rag^−/−^ mice were originally obtained from the mouse colony of Drs. Diane Mathis and Christophe Benoist at the Jackson Laboratory. B6 mice were originally obtained from the Jackson Laboratory. All experimental mice were bred in-house and maintained in the University of Arizona SPF Barrier Facility until removal to a BSL-2 mouse room prior to infection. All animal experiments were approved by the University of Arizona Institutional Animal Care and Use Committee.

### Microbiota reconstitution and quantification

SFB colonization was described previously (Teng et al., 2016). Briefly, mice were maintained SFB(−) unless SFB(+) status was necessary. SFB was originally introduced to our mouse colony by gavaging mice with SFB-containing feces from B6 mice purchased from Taconic Biosciences; thereafter, mice were gavaged with SFB-containing feces collected from the SFB(+) mice in our colony. To obtain SFB(+) groups for analysis, mice were weaned at 21 days and rested for 1 day, then orally gavaged for 3 consecutive days with 200 μl each day of a solution made from fecal pellets mashed in a 0.9% NaCl solution, then filtered through 100 μm nylon mesh. SFB(−) mice for the same experiments were ungavaged littermates or age-matched controls. The SFB colonization status was examined by SFB-specific 16S rRNA quantitative PCR (Wu et al., 2010) at 10 days after the first day of gavage.

### NP-ficoll immunization

Mice were immunized i.p. with 200 μl of a 1:1 mixture of 100 μg NP(49)-AECM-Ficoll (Biosearch Technologies, Inc.) and Imject Alum (Fisher). 6 days later, mice were euthanized for analysis.

### ELISAs

ELISA plates were coated with 5 μg/ml NP-BSA, at a conjugation ratio of 4 or 49, or 10^8^ heat-inactivated CFU-equivalents/ml overnight. Mouse serum was diluted 1:500 (for α-NP IgM detection) or 1:100 (for α-NP IgG3) or 1:50 (for α-*S. pneumoniae* detection) and added to plate, then diluted serially. Subsequently, plates were washed and alkaline phosphatase-conjugated anti-mouse IgM or IgG3 was added. After the final wash, alkaline phosphatase substrate was added and optical density at 405 nm was read using a VersaMax Tunable microplate reader (Molecular Devices). Titer was calculated as described previous (Teng et al., 2016). Briefly, ab titer was expressed as arbitrary units, calculated as the reciprocal of the highest dilution at which the OD reached 0.15 (twice the background level).

### Murine pneumonia model

For intranasal infections, mice were anesthetized by i.p. injection of King's Cocktail (7.5 mg/ml Ketamine, 0.5 mg/ml Xylazine in sterile DPBS, obtained separately from Western Medical Supply). 20 μl of the bacterial suspension was inoculated slowly into each nare (40 ul total/mouse). Mice were observed for signs of illness (hunched posture, ruffled fur/lack of grooming, lethargy, labored breathing) and measured for temperature daily until euthanasia at indicated time points. Disease scores were assigned based on symptoms, i.e., 1 = no disease; 2 = hunching, ungroomed coat; 3 = lethargic; 4 = moribund. Temperature was measured daily beginning at day of infection (D0) using a non-contact infrared thermometer (Maxsio). The thermometer is aimed at the inside of the mouse's left ear. A drop in temperature of >6°C compared to the initial temperature or a weight loss of >20% was considered moribund, and the mouse was euthanized. Mice were euthanized by CO_2_ inhalation.

### Bacterial counts from tissues

Spleens and lungs were collected and weighed. The middle lobe (on the right side) of the lung and half of the spleen were placed in new tubes of PBS, weighed, and homogenized using a PowerGen 125 homogenizer (Fisher). Serial 10-fold dilutions of the homogenates in PBS were plated onto TSA-II plates with 5% sheep blood and incubated overnight at 37°C with 5% CO_2_. Colonies were then counted and CFU per total organ was calculated.

### Heat-inactivated bacterial challenge

*Streptococcus pneumoniae* ATCC 6303 was grown to an OD_600_ of 0.4–0.6, then heat-inactivated by incubating in a 56°C water bath for 2 h. Aliquots were plated before and after heat inactivation to confirm bacterial density and proper inactivation of bacteria. Heat-inactivated bacteria were stored at −80°C until use. 10^8^ CFU equivalents were administered intranasally as described above, at 2 time points 12 h apart. Mice were euthanized and tissues analyzed 3 days after the initial treatment.

### CD47 *in Vivo* blocking experiment

400 μg anti-CD47 (clone MIAP410, BioXCell) or control IgG (Jackson Immunoresearch) in 200 μl DPBS was administered i.p. 1 day before infection. Mice were infected and monitored as described above.

### Adoptive transfer

Splenic CD19^+^ B cells were enriched by MACS enrichment (Miltenyi) or EasySep enrichment (StemCell) from B6 mice, and 5 × 10^6^ B cells were adoptively transferred by retro-orbital (r.o.) injection into SFB(−) Rag^−/−^ recipients, anesthetized using King's Cocktail (described above). Mice were rested for 2 weeks to allow B cell reconstitution before infection.

### Lung single cell preparation

Lung single cell preparation was described previously (Naskar et al., 2017). Briefly, lungs were perfused through the right ventricle of the heart with 10 ml of PBS to flush blood, then finely minced. Minced lung was transferred to a 50 ml tube containing 10 ml of digestion buffer [1 mg/ml Collagenase D (Roche), 1 mg/ml MgCl_2_ (Fisher), and 0.1 mg/ml DNase I (Sigma) in DMEM (HyClone)]. Lungs were digested for 25–30 min at 37°C with rotation (200 rpm), then passed through a 100 μm cell strainer. The digestion tubes were washed with 5 ml PBS, which was then poured through the cell strainer. A plunger from a 5 ml syringe was then used to grind the remaining tissue pieces on the cell strainer, which were then washed with 5 ml of PBS with 1% EDTA (Fisher). Lung suspension was centrifuged down and resuspended in DMEM-C (DMEM supplemented with 10% FCS, 1% nonessential amino acids, penicillin, streptomycin, and glutamine and 0.1% 2-mercaptoethanol).

### Antibodies and flow cytometry

Lung and spleen cells were incubated with Fc block (supernatant from 2.4G2 cells, with 0.05% sodium azide) for 20 min at 4°C, then washed with PBS and stained with Live/Dead Fixable Yellow (Invitrogen) for discrimination of dead cells. For surface staining, fluorophore-conjugated mAbs specific for CD45 (30-F11), CD11b (M1/70), Ly6G (1A8), CD62L (MEL-14), CD18 (M18/2), and CD47 (miap301) were obtained from BioLegend. Cells were fixed using a Cytofix/Cytoperm kit (BD Biosciences), and run on an LSR II (BD Biosciences), and analyses were performed with FlowJo (TreeStar) software.

### Histology

The chest cavity was opened, ribs were cut away, and the trachea was exposed and cannulated with the flexible plastic sheath from a 20 g safelet catheter (Exelint International). After removal of the right lobes of the lung for flow cytometry analysis, 5 ml of 10% buffered formalin (Fisher) was injected slowly over the course of a few minutes through the left bronchus into the left lung. The left lung was then removed and placed in 5 ml of 10% formalin and allowed to fix for 24 h, after which it was transferred to 70% EtOH. Paraffin embedding and sectioning was performed by the University of Arizona Tissue Acquisition and Cellular/Molecular Analysis Shared Resource. 5 μm sections were deparaffinized in xylenes, rehydrated, immersed in hematoxylin (VWR) solution for 30 s, rinsed with distilled water, differentiated in acetic alcohol, immersed in eosin (VWR) solution for 5 s, dehydrated through a graded ethanol series, cleared, and coverslipped. One lung section per mouse was analyzed. Under a digital VHX-6000 light microscope (Keyence), the whole section of lung tissue was stitched from images taken with a 4× objective. Using ImageJ (NIH) analysis software, the number of foci, the mean area of foci, the total area of foci, and the percentage area of foci covering each section were assessed by manually counting and tracing the area of foci. Data are expressed as mean ± standard error of mean (SEM). Statistical analyses were performed with Prism 6.0 software (GraphPad), with the level of significance set at *p* < 0.05. Differences between experimental groups were analyzed using a Student's *t*-test.

### RT-QPCR

Lung tissue from infected or uninfected mice was collected into Tripure Isolation Reagent (Roche). RNA was extracted according to the manufacturer instructions, with the addition of an RNA purification step after resuspension in DEPC-treated ddH_2_O. Briefly, 0.1 volume of sodium acetate (Fisher) was added to each sample, followed by 2.5 volume of 100% Pure Ethyl Alcohol, 200 proof, Molecular Biology Grade (Sigma). The samples were mixed thoroughly, incubated at −80°C for 20 min, then centrifuged at 12000 rpm for 10 min at 4°C. After this step, supernatant was decanted, samples were washed with 70% Ethanol in DEPC-treated ddH_2_O, centrifuged at 9100 rpm for 5 min at 4°C, and dried in a biosafety cabinet. First-strand cDNA synthesis was performed using the Maxima First Strand cDNA Synthesis Kit (Fisher) according to manufacturer instructions. cDNA was detected using Taqman Fast Advanced Master Mix (Fisher) and the following Taqman Gene Expression Assays: HPRT (Mm01545399_m1); IL-1β (Mm00434228_m1); IL-6 (Mm00446190_m1); IL-12a (Mm00434165_m1); and IFNγ (Mm01168134_m1). Data for individual cytokines were normalized to HPRT, followed by normalization to an uninfected, SFB(−) Rag^−/−^ individual.

### Statistics

All analyses were performed using GraphPad Prism. Differences were considered significant when *p* < 0.05 by two-tailed, unpaired *t*-test with Welch's correction. To calculate differences in temperature change over the course of infection, the area under the curve was calculated for each mouse within an experimental group, followed by the *t*-test described above to compare area under the curve between groups. One asterisk (^*^) indicates *p* < 0.05, two asterisks (^**^) indicate *p* < 0.01, and three asterisks (^***^) indicate *p* < 0.001.

## Results

### SFB enhances the systemic T-independent II response

**Figure 1:**
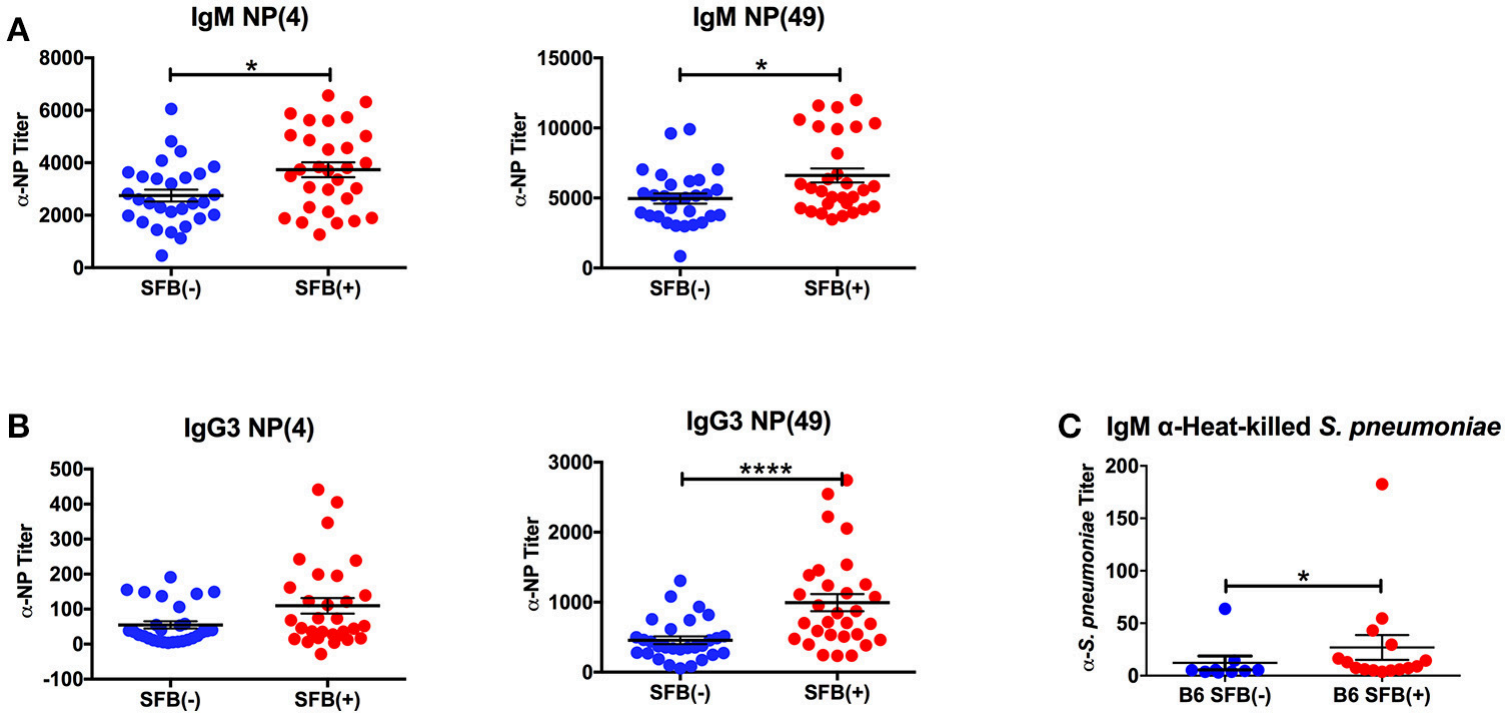
SFB enhances the TI-II response. TCRα^−/−^ mice were immunized i.p. with NP-Ficoll plus alum, then serum was collected 6 days later for ELISA analysis. Symbols represent individual animals, and bars represent the means ± standard error. **(A,B)** ELISAs on serum from NP-Ficoll-immunized SFB(−) and SFB(+) mice. Plate coated with indicated NP conjugate. (*N* = 29–30/group). **(C)** ELISA against heat-killed *S. pneumoniae* using serum from SFB(−) and SFB(+) B6 mice infected intranasally with 1 × 10^6^ CFU for 3 days. Data are compiled from 5 independent experiments (*N* = 9–15/group). ^*^p > 0.05; ^****^p > 0.0001.

We began by studying the role of the microbiota in a more traditional immunocompetent host using the wild type B6 mouse strain. Adaptive immune protection against infection with *S. pneumoniae* is based mainly on the generation of antibodies to pneumococcal capsular polysaccharides, prototypical TI-II antigens (Caya et al., 2015). In order to determine whether SFB is capable of enhancing TI-II antibody production without the help of cognate T cells, we immunized SFB negative or SFB colonized [SFB (−) and (+) hereafter] mice lacking conventional T cells (TCRα^−/−^ mice) with NP-Ficoll, a model TI-II antigen. Because TI-II antigens robustly induce antibodies in the IgM and IgG3 isotypes (Swanson et al., 2010), we measured the serum antibody response to NP-Ficoll in the IgM and IgG3 isotypes by ELISA 6 days after immunization. Additionally, high affinity anti-NP antibodies were measured by coating the ELISA plates with NP-bovine serum albumin (NP-BSA) with a low ratio (4:1) of NP epitopes to protein conjugate while both low and high affinity anti-NP antibodies were detected with a high ratio of NP to protein conjugate (49:1) (Swanson et al., 2010). SFB(+) mice had higher anti-NP IgM and IgG3 responses compared to their counterparts that lacked SFB (Figures 1A,B). We found that while SFB enhanced both high and low-affinity antibodies of the IgM isotype, it had a greater effect on low-affinity antibodies of the IgG3 isotype than on high-affinity IgG3 antibodies (Figure 1B). These results suggest that SFB boosts TI-II antibody production in the absence of conventional T cells.

Because SFB boosted the antibody response to NP-Ficoll, we next determined whether it would similarly affect the response to a microbe-derived TI-II antigen, the encapsulated *S. pneumoniae* (Chiavolini et al., 2008). Using a serotype 3 strain of *S. pneumoniae* originally derived from a clinical isolate, we infected SFB(−) and SFB(+) C57BL/6 (B6) mice intranasally with a bacterial dose of 1 × 10^6^ CFU. We measured the antibody response 3 days post infection, at which point the infection begins to resolve in immunocompetent B6 mice (Bou Ghanem et al., 2015). We found that during acute pneumonia induced by *S. pneumoniae*, SFB colonization was associated with a higher anti-*S. pneumoniae* IgM titer (Figure 1C). As this difference was observed at 3 days post infection, it may reflect the beginning of the polysaccharide-specific antibody response (Colino and Outschoorn, 1998), perhaps in combination with natural antibodies, some of which react with phosphorylcholine, which can be found on *S. pneumoniae* (Khan et al., 2004). We observed very little, if any, IgG3 induction at this time point (data not shown). Thus, SFB can enhance antibody production against a TI-II antigen during an infection.

### SFB-enhanced TI-II responses do not provide additional protection against *S. pneumoniae* in immunocompetent hosts during the acute phase of infection

**Figure 2:**
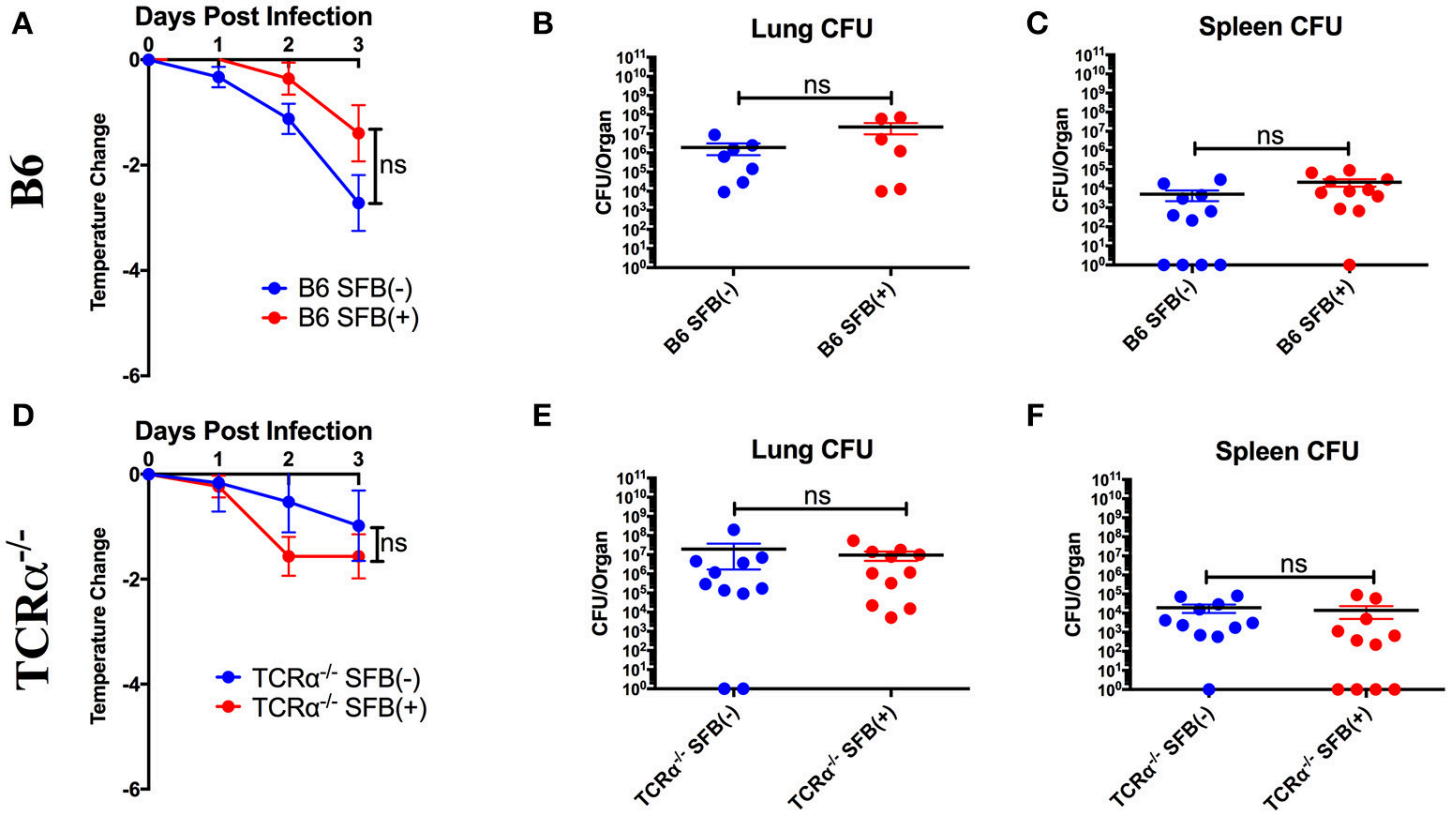
SFB does not add to protection against *S. pneumoniae* lung infection for immunocompetent or TCRα^−/−^ mice during the acute phase of infection. B6 or TCRα^−/−^ mice were infected intranasally with *S. pneumoniae*, and infection course was followed over 3 days. **(A)** Change in temperatures of SFB(−) and SFB(+) B6 mice (*N* = 18-19/group, 5 assays combined). **(B,C)** CFU in lungs **(B)** or spleen **(C)** of SFB(−) and SFB(+) B6 mice at 3 days post infection (*N* = 6-11/group, 2-3 assays combined). **(D)** Change in temperatures of SFB(−) and SFB(+) TCRα^−/−^ mice (*N* = 11–12/group, 3 assays combined). **(E,F)** CFU in lungs **(E)** or spleen **(F)** of SFB(−) and SFB(+) TCRα^−/−^ mice at 3 days post infection (*N* = 11/group, 3 assays combined). Bars represent the means ± standard error.

Based on the ability of SFB to boost antibodies against *S. pneumoniae*, we tested the hypothesis that SFB colonization would confer greater protection against *S. pneumoniae* infection, as mice deficient in production of antigen-specific TI-II antibodies against *S. pneumoniae* displayed a severe defect in protection upon infection (Haas et al., 2005). Protection was determined by change in temperature, which is a more reliable method of determining likelihood of mortality than weight loss in *S. pneumoniae* infection (Trammell and Toth, 2011). However, when we infected immunocompetent B6 mice, we found that there was no significant protection conferred by SFB colonization against body temperature reduction during the acute phase of infection (Figure 2A). To confirm that the similar temperature drops were associated with equivalent bacterial control, we examined bacterial colony forming units (CFU) in the lungs, the initial site of infection (Figure 2B). We also examined systemic bacterial load using the spleen, because *S. pneumoniae* is known to cause bacteremia by escaping the lungs and transmigrating into the blood, from which it reaches the spleen, after about 24 h of infection (Bhowmick et al., 2013). We found that bacterial burdens in the lungs and spleen were comparable between the SFB(−) and SFB(+) groups (Figures 2B,C). From these data, we concluded that SFB is not able to provide any additional boost in protecting immunocompetent hosts when challenged by *S. pneumoniae* acute lung infection.

### SFB enhances the resistance against *S. pneumoniae* acute infection in immunocompromised hosts

As *S. pneumoniae* infections are most dangerous for immunocompromised individuals, we decided to set up an animal model that could mimic the severe disease phenotype in immunocompromised individuals by systemically targeting immune component(s), and address whether the gut microbiota exerts a protective effect in these immunocompromised hosts. To test this, we first examined the potential for SFB-mediated protection in the absence of conventional T cells by infecting TCRα^−/−^ mice (lacking αβ T cells). We found that TCRα^−/−^ mice were not impaired in resistance to *S. pneumoniae* compared to WT mice regardless of SFB colonization status (Figures 2D–F, compare Figures 2A–C). These data suggested that T cell deficiency did not render the host more susceptible to *S. pneumoniae* infection compared to the WT condition, and SFB did not provide additional protection against acute *S. pneumoniae* lung infection in animals lacking αβ T cells, similar to what we observed in WT mice.

**Figure 3:**
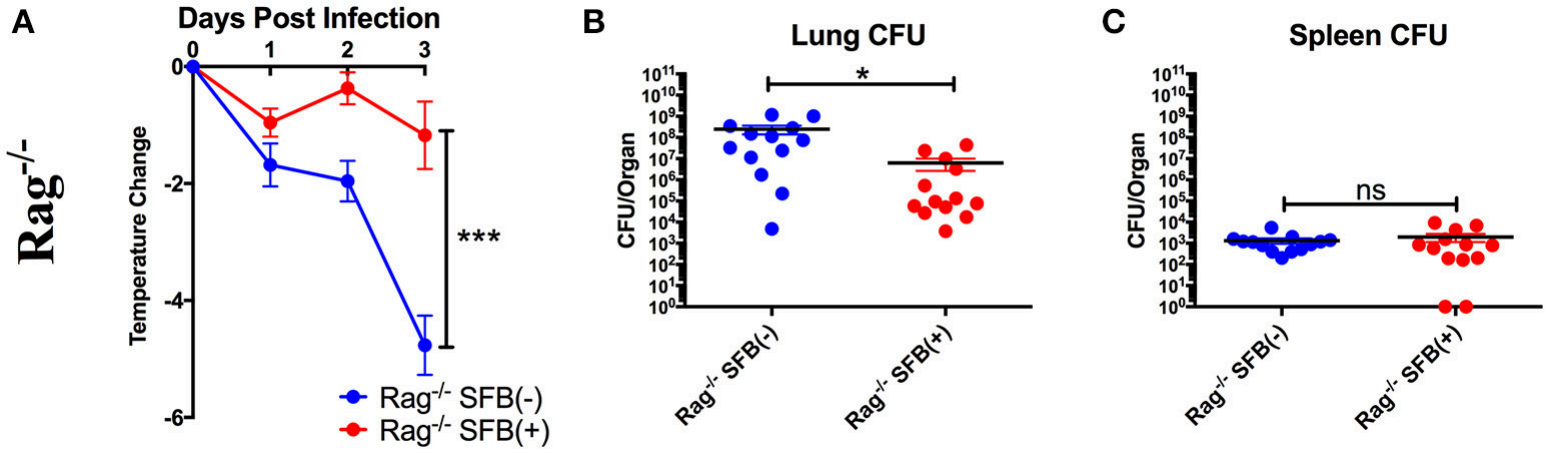
SFB enhances the resistance against *S. pneumoniae* acute lung infection in adaptive immune-deficient mice. **(A–C)** Rag^−/−^ mice were infected intranasally with *S. pneumoniae*, and infection course was followed over 3 days. **(A)** Change in temperatures of SFB(−) and SFB(+) Rag^−/−^ mice (*N* = 17/group, 5 assays combined). **(B,C)** CFU in lungs **(B)** or spleen **(C)** of SFB(−) and SFB(+) Rag^−/−^ mice at 3 days post infection (*N* = 13/group, 4 assays combined). Bars represent the means ± standard error. ^*^p < 0.05, ^***^p < 0.001.

Next, we tested SFB-mediated protection in hosts deficient in adaptive immunity by infecting SFB(−) and SFB(+) Rag^−/−^ mice, which lack both T and B cells, and thus are entirely dependent on innate immunity for protection. When we compared the change in temperature and bacterial burden between SFB(−) Rag^−/−^ and WT mice, we found that the Rag^−/−^ group has a severe temperature drop compared to the WT group (Figure 3A, compare Figure 2A). This correlates with a higher bacterial burden in the lungs, ~70 fold higher in Rag^−/−^ mice than in WT mice. Strikingly, SFB colonization was able to rescue Rag^−/−^ mice from this severe temperature loss. Thus, unlike their SFB(−) counterparts, SFB(+) Rag^−/−^ mice had surprisingly stable temperatures throughout the infection, and lower bacterial burdens in the lungs, similar to what we observed in WT mice (Figures 3A–C). This suggested that SFB was able to provide additional help against *S. pneumoniae* infection by modulating innate immunity in the deficiency of adaptive immunity. To confirm that the effects we saw were not due to better colonization of SFB in Rag^−/−^ compared to WT mice, we compared the relative SFB levels in each group. Rag^−/−^ mice turned out to have a slightly lower level of SFB colonization than WT mice, confirming that the SFB-mediated protection we observed in Rag^−/−^ mice was not simply the result of increased SFB (Supplementary Figure 1). We had thus far used a dose of *S. pneumoniae* of 7 × 10^4^−1 × 10^5^ CFU in our bacterial challenge. We next set out to push the limits of the system and determine whether SFB colonization would still be beneficial at a higher dose of *S. pneumoniae* at 1–1.5 × 10^6^ CFU. We found that even at this higher dose, SFB was still able to enhance resistance, as we observed that the SFB(+) group had more stable temperatures and lower bacterial burdens than the SFB(−) group (Supplementary Figures 2A–C).

**Figure 4:**
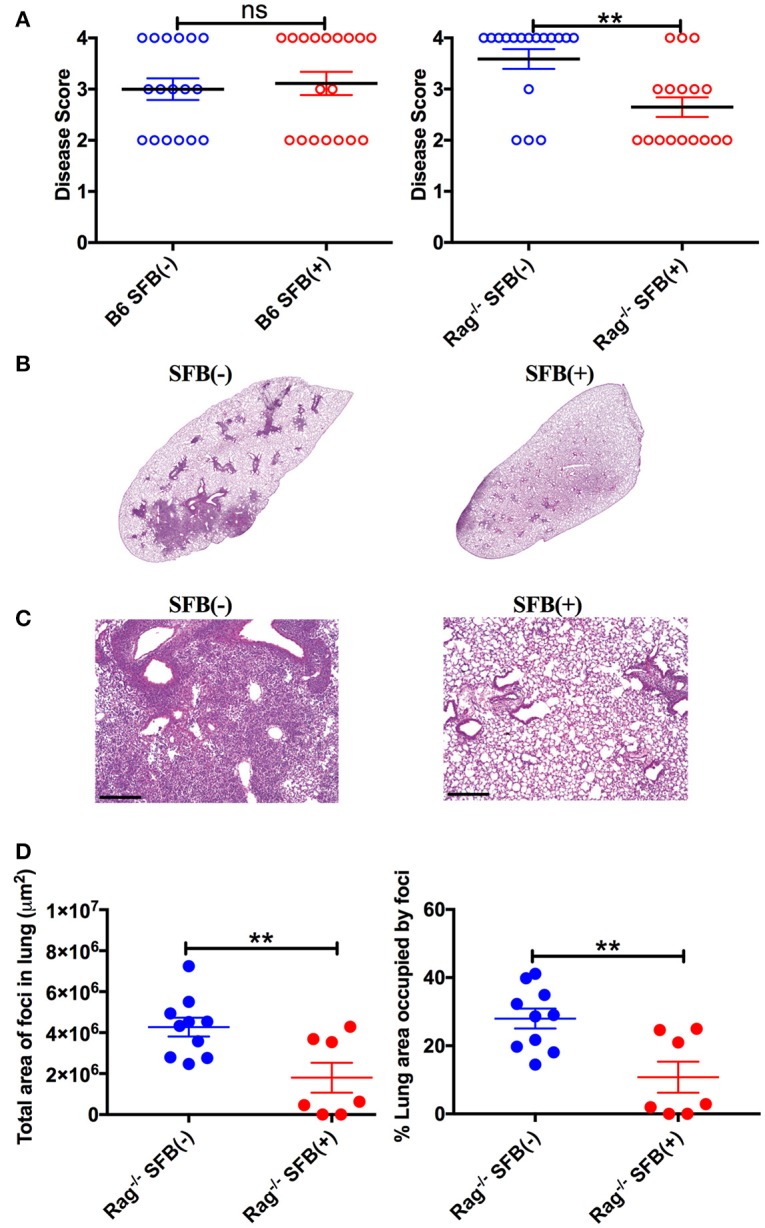
Disease scores and histology reflect positive impact of SFB colonization only in adaptive immune-deficient mice, but not immunocompetent mice. **(A)** B6 or Rag^−/−^ mice were infected intranasally with *S. pneumoniae*, and infection course was followed over 3 days. At 3 days post infection, mice were scored for signs of illness as detailed in Methods. **(B–D)** Rag^−/−^ mice were infected intranasally with *S. pneumoniae*. Lung sections of left lobe of lung from SFB(−) and SFB(+) Rag^−/−^ mice at 3 days post infection were stained with H&E and examined by light microscopy. **(B)** Representative images. Original magnification 4X. **(C)** Representative images. Original magnification 10X. Scale bar = 250 μm. **(D)** Quantification of total area and percentage of total lung covered by foci of leukocyte infiltration. Bars represent the means ± standard error. ^**^p < 0.01.

To further characterize SFB-mediated host resistance against *S*. *pneumoniae* infection, we determined the sickness of infected Rag^−/−^ mice using a disease scoring system with 4 indicating the most severe disease and 1 indicating no disease, modified from the Morton and Griffiths scale as described by De Filippo et al. (2014). We found that as indicated by the body temperature data, there is no difference in disease score between SFB(−) and (+) WT mice. In contrast, Rag^−/−^ mice in the absence of SFB colonization developed severe disease, while SFB colonization ameliorates the disease in Rag^−/−^ mice (Figure 4A). Furthermore, histological analysis of lung sections showed less lung damage and decreased leukocyte infiltration in SFB(+) Rag^−/−^ mice compared to SFB(−) mice (Figures 4B–D). Thus, the gut commensal SFB provided much-needed resistance against *S. pneumoniae* lung infection in immunocompromised hosts.

### B cells contribute to protection against *S. pneumoniae* infection regardless of SFB colonization status

**Figure 5:**
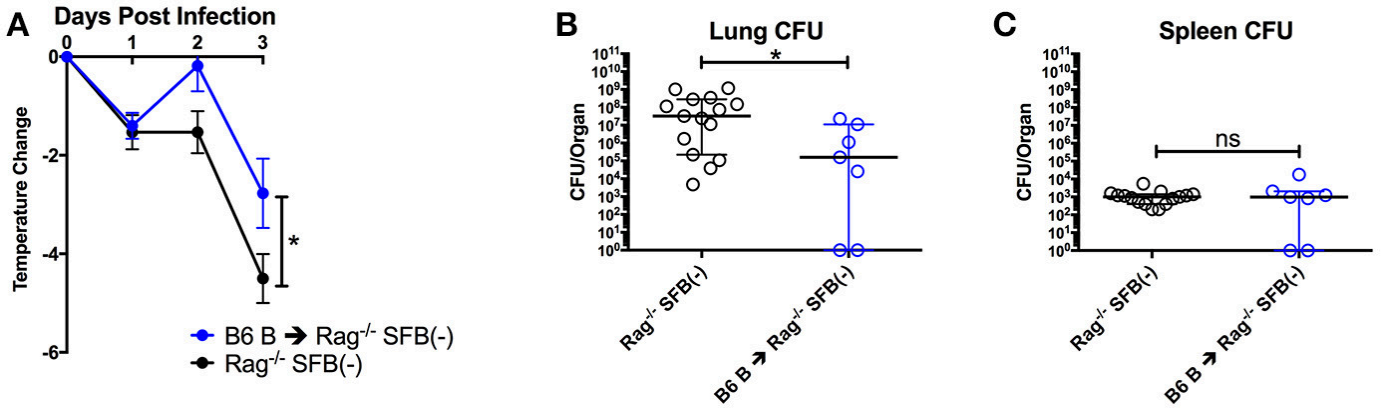
B cells contribute to protection against *S. pneumoniae* infection regardless of SFB colonization. **(A–C)** Mice were infected intranasally with *S. pneumoniae*, and infection course was followed over 3 days. **(A)** Change in temperatures of SFB(−) Rag^−/−^ mice either transferred with 5 × 10^6^ B6 B cells or DPBS (*N* = 7–19/group, 9 assays combined). **(B,C)** CFU in lungs **(B)** or spleen **(C)** of SFB(−) Rag^−/−^ mice either transferred with 5 × 10^6^ B6 B cells or DPBS (*N* = 10–15/group, 9 assays combined). Bars represent the means ± standard error. ^*^p < 0.05.

When taking a closer look at the difference in SFB enhancement of resistance between the Rag^−/−^ and WT mice, we noted one major difference: SFB(−) Rag^−/−^ mice had a more severe drop in body temperature and higher bacterial burdens in the lungs when infected with *S. pneumoniae* than did WT mice Figures 2A,B,3A,B). This observation sparked our interest in determining what missing immune component causes the difference between WT mice and Rag^−/−^ mice in the SFB(−) condition upon challenge with *S. pneumoniae*. Because TCRα^−/−^ mice had limited loss in body temperature, similar to WT mice, we concluded that the severe body temperature drop in SFB(−) Rag^−/−^ mice was not due to a lack of conventional T cells. As Rag^−/−^ mice lack both T and B cells, we thus hypothesized that B cells are responsible for the increased protection against *S. pneumoniae* lung infection in WT compared to Rag^−/−^ mice. To directly address this, we transferred B cells into SFB(−) Rag^−/−^ recipients, allowed 2 weeks for reconstitution of the B cell population, and compared transfer recipients' response to infection to mice that received PBS alone. We found that B cell transfer is sufficient to rescue SFB(−) Rag^−/−^ mice from severe body temperature loss, as our data showed that similar to WT mice, the SFB(−) Rag^−/−^ mice receiving B cells only displayed a mild temperature drop, which is accompanied by a lower lung bacterial burden than those that did not receive B cells (Figures 5A,B). As seen earlier, there was no change in splenic bacterial burden between the two conditions (Figure 5C). These findings demonstrate that B cells contribute to resistance to *S. pneumoniae* lung infection in an SFB-independent manner.

### SFB colonization promotes neutrophil resolution in the lungs of immunodeficient rag^−/−^ hosts during the inflammation resolution phase

**Figure 6:**
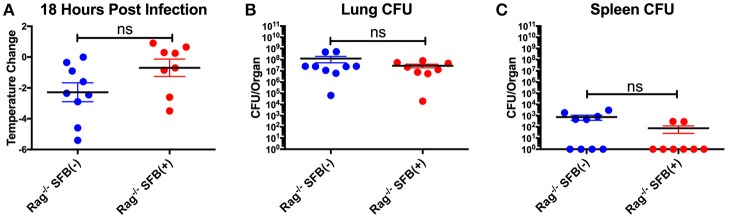
Lack of significant differences in temperature and CFU between SFB(−) and SFB(+) Rag^−/−^ mice at 18 h post infection. **(A–C)** Rag^−/−^ mice were infected intranasally with *S. pneumoniae*. At 18 h post infection, mice were analyzed for temperature loss **(A)** and bacterial burden in the lungs **(B)** and spleen **(C)**. (*N* = 8–9/group, 3 assays combined). Bars represent the means ± standard error.

**Figure 7:**
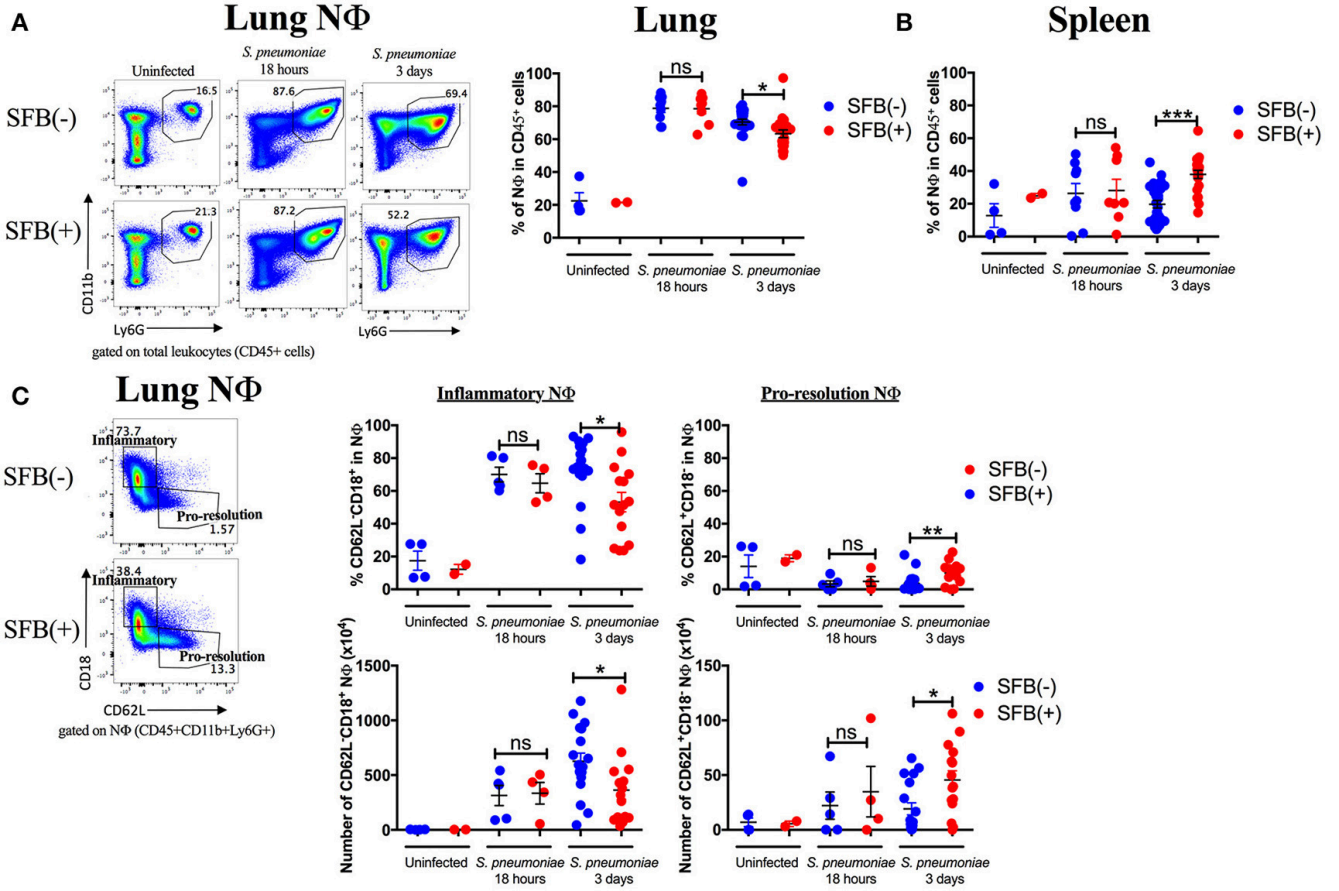
SFB-mediated enhanced resistance corresponds with increased neutrophil resolution in the lung. Mice were infected intranasally with *S. pneumoniae*. **(A,B)** Representative plots and compiled graphs of neutrophil (CD11b^+^Ly6G^+^) frequency as a percentage of total leukocytes (CD45^+^) in Rag^−/−^ lungs **(A)** and spleens **(B)** uninfected (*N* = 2–4/group, 2 assays combined) or at 18 h post infection (*N* = 8–9/group, 3 assays combined) and 3 days post infection (*N* = 21–25/group, 7 assays combined). **(C)** Frequency (as a percentage of total neutrophils) and numbers of inflammatory (CD62L^−^CD18^+^) or pro-resolution (CD62L^+^CD18^−^) neutrophils in Rag^−/−^ lungs uninfected or at 18 h post infection (*N* = 2–9/group, 3 assays combined) and 3 days post infection (*N* = 15–17/group, 5 assays combined). Bars represent the means ± standard error. ^*^p < 0.05, ^**^p < 0.01, ^***^p < 0.001.

Next, we aimed to address the SFB-mediated immunomodulation that may play a role in SFB-mediated innate immune resistance against *S. pneumoniae* infection in the lungs of Rag^−/−^ mice. We first examined the lung neutrophil populations that are crucial for defense against *S. pneumoniae* infections (De Filippo et al., 2014). Although we originally observed SFB-mediated resistance against lung infection at day 3 post infection (Figure 3), we decided to extend our model to examine an earlier time point, 18 h post infection, a time point when neutrophils are required for host survival during *S. pneumoniae* infection (Bou Ghanem et al., 2015). At 18 h post infection, we observed a trend toward prevention of temperature loss but no significant change in temperature or bacterial burden in the lungs or spleen (Figures 6A–C). When we examined the neutrophil population at this time point, there was a huge influx of neutrophils into the lungs, as there was a dramatic increase of lung neutrophil percentage in infected (60–90%) compared to uninfected animals (~20%) (Figure 7A). Yet there was no difference in neutrophil frequency or numbers between the infected SFB(−) and (+) spleen or lung tissues at 18 h post infection (Figures 7A,B, Supplementary Figure 3A). At 3 days post infection, there is overall a trend toward a decrease in lung neutrophil frequency, which corroborated an earlier report indicating day 3 begins the resolution phase of *S. pneumoniae* infection (Bou Ghanem et al., 2015). Importantly, unlike at the time point of 18 h post infection, we observed a difference between the SFB(−) and (+) groups, as our data showed a decrease in neutrophil percentages as well as absolute numbers in the lungs of infected SFB(+) Rag^−/−^ mice compared to their SFB(−) counterparts (Figure 7A, Supplementary Figure 3A). We also observed an increase in neutrophil frequency in the spleen (Figure 7B), which may indicate an increase in emergency granulopoiesis, which is induced during infections and increases the output of neutrophils and monocytes (Bronte and Pittet, 2013; Boettcher and Manz, 2017). The decrease in lung neutrophils at 3 days post infection brought up the question of whether the neutrophils present in SFB(+) lungs have a pro-resolution phenotype and represent a neutrophil population that is returning to homeostatic conditions. Activated inflammatory neutrophils express different levels of integrins and other immune-related molecules that are crucial for their functional activity compared to pro-resolution neutrophils. Important molecular changes include L-selectin (CD62L), which decreases during activation, allowing the cell to stop rolling in blood vessels, while CD18 (integrin β2) increases in activated inflammatory neutrophils, allowing cells to create firm adhesions to the endothelium and eventually transmigrate from the blood to the tissues (Simon et al., 1995; Toledo et al., 2009; Falcao et al., 2015; Campbell et al., 2016).

Following the earlier observation that there was no change in neutrophil frequency between SFB(−) and (+) Rag^−/−^ mice at 18 h post infection, we found that there may be a very slight trend but no significant change in either the inflammatory (CD62L^−^CD18^+^) or pro-resolution (CD62L^+^CD18^−^) neutrophil phenotype at 18 h post infection (Figure 7C). In contrast, at day 3 post infection when there is a significant drop in the lung neutrophil population in SFB(+) compared to SFB(−) Rag^−/−^ mice, we found that there was a reduction in lung neutrophils with the inflammatory phenotype in SFB(+) Rag^−/−^ mice (Figure 7C). Along with this decrease in the inflammatory phenotype, there was an increase in CD62L expression, and a decrease in CD18 expression, on lung neutrophils from SFB(+) Rag^−/−^ mice, corresponding to a less activated, pro-resolution neutrophil phenotype (Figure 7C; Simon et al., 1995; Toledo et al., 2009; Falcao et al., 2015; Campbell et al., 2016). There was no significant change in neutrophil phenotype at either infected time point in the spleen (Supplementary Figure 3B). To better understand the inflammatory milieu in the lung at day 3 post infection, when we observed the SFB-mediated resistance against bacterial infection, we also examined cytokine mRNA expression levels in the lungs including IL-1β, IFNγ, IL-12a, and IL-6, all of which have been demonstrated to be induced in the lungs during *S. pneumoniae* infection (Bergeron et al., 1998; Mcneela et al., 2010; Supplementary Figure 4). We found no change in IL-1β or IFNγ, but a decrease in IL-12a and a trend toward a decrease in IL-6. These data suggest that not only are there fewer neutrophils in the lungs of SFB(+) Rag^−/−^ mice at 3 days post infection, but those present are also in a less inflammatory, more pro-resolution state than those found in SFB(−) Rag^−/−^ mice. These data were further supported by the less inflammatory cytokine milieu in the SFB(+) compared to the SFB(−) group. We also examined the impact of SFB on other innate cell types involved in the immune response against *S. pneumoniae* infection, including alveolar macrophages, dendritic cells, and monocytes in the lungs (Zhang et al., 2000; Kirby et al., 2006; Daigneault et al., 2011; Dockrell et al., 2012), and found no difference in the percentage of these cell types in SFB(−) vs. SFB(+) Rag^−/−^ mice at 3 days post infection (data not shown), and thus decided to further focus on and investigate SFB-modulated neutrophil responses.

### SFB-mediated protection corresponds with a decrease in a “don't eat me signal,” CD47, on inflammatory lung neutrophils

**Figure 8:**
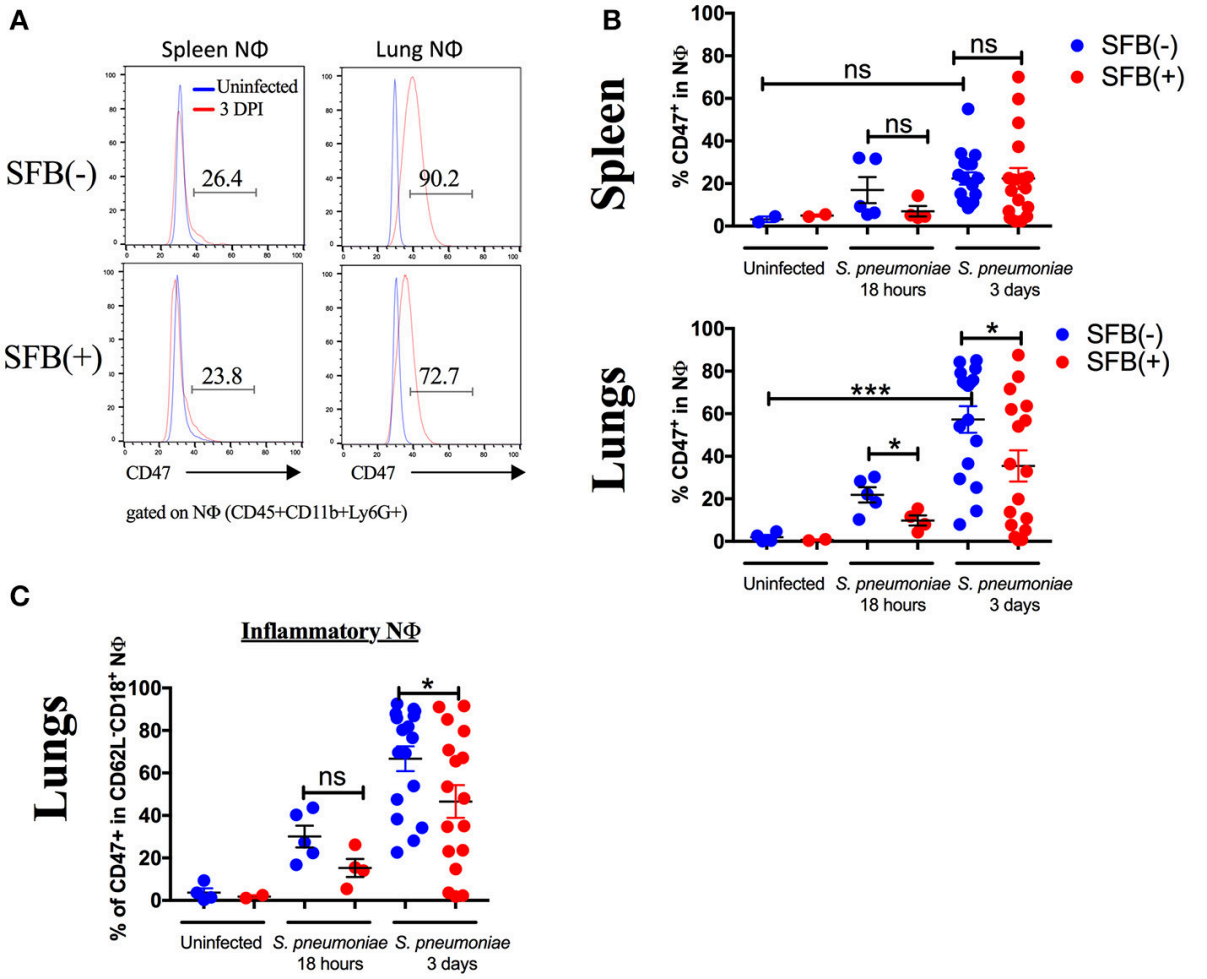
SFB colonization is associated with decreased expression of CD47 in inflammatory neutrophils in the lung. **(A)** Representative histograms of CD47 expression in neutrophils in uninfected (blue) or 3 days infected Rag^−/−^ mice (red) in SFB(−) and SFB(+) lungs and spleen, as indicated. Numbers shown indicate frequency of CD47+ neutrophils as a percentage of total neutrophils in infected mice 3 days post infection. **(B)** Frequency of spleen or lung neutrophils expressing CD47 as a percentage of total neutrophils in Rag^−/−^ mice either uninfected or at 18 h or 3 days post infection (Uninfected *N* = 2–4/group, 2 assays combined; 18 h post infection *N* = 8–9/group, 3 assays combined; 3 days post infection *N* = 17/group, 5 assays combined). **(C)** Frequency of inflammatory lung neutrophils expressing CD47 as a percentage of total inflammatory lung neutrophils in Rag^−/−^ mice either uninfected or at 18 h or 3 days post infection (Uninfected *N* = 2–4/group, 2 assays combined; 18 h post infection *N* = 8–9/group, 3 assays combined; 3 days post infection *N* = 17/group, 5 assays combined). Bars represent the means ± standard error. ^*^p < 0.05, ^***^p < 0.001.

Neutrophils are proficient phagocytes and antimicrobial effectors, but their effector functions are non-specific and can cause damage to the surrounding tissue (Fujie et al., 1999; Saffarzadeh et al., 2012). Therefore, down-regulating neutrophil numbers and returning them to the basal level is essential for successful disease resolution. The less inflammatory phenotype of the lung neutrophil population in SFB(+) Rag^−/−^ mice brings up the possibility that these neutrophils may be associated with pro-resolution activity. Thus, we next set out to determine the role of SFB in decreasing the lung neutrophil number and inflammatory state in immunocompromised hosts. We hypothesized that in SFB(+) Rag^−/−^ mice, the lung inflammatory neutrophils were experiencing enhanced clearance by phagocytes, a process termed efferocytosis (Buckley et al., 2006). To test this, we examined whether there is a reduction, in SFB(+) animals, in surface expression of the “don't eat me” molecule CD47, an integrin-associated protein that binds to SIRPα on phagocytes to inhibit efferocytosis (Barclay and Van den Berg, 2014), which might explain the enhanced reduction in the lung neutrophil population in SFB(+) Rag^−/−^ mice. Compared to the uninfected group, *S. pneumoniae* lung infection in general significantly increased CD47 expression in lung neutrophils, and to a lesser extent in splenic neutrophils (Figures 8A,B). Notably, SFB colonization significantly decreased CD47 expression on infected lung neutrophils as early as 18 h post infection and continued through 3 days post infection (Figures 8A,B, Supplementary Figure 5). At 3 days post infection, this was driven by a decrease in CD47 specifically on inflammatory lung neutrophils (Figure 8C, Supplementary Figure 5). CD47 expression at 18 h post infection follows a similar trend (Figure 8C). This suggests that inflammatory neutrophils in the lungs of SFB(+) Rag^−/−^ mice might be more susceptible to efferocytosis than their counterparts in SFB(−) Rag^−/−^ mice. Thus, the decreased numbers of inflammatory lung neutrophils corresponded with the decrease in CD47 expression in SFB(+) compared to SFB(−) Rag^−/−^ mice, suggesting that SFB may promote greater neutrophil efferocytosis, leading to better resolution of inflammatory neutrophils in the lungs of immunocompromised Rag^−/−^ mice during *S. pneumoniae* infection.

### SFB modifies lung neutrophil phenotype independent of *S. pneumoniae* bacterial clearance

**Figure 9:**
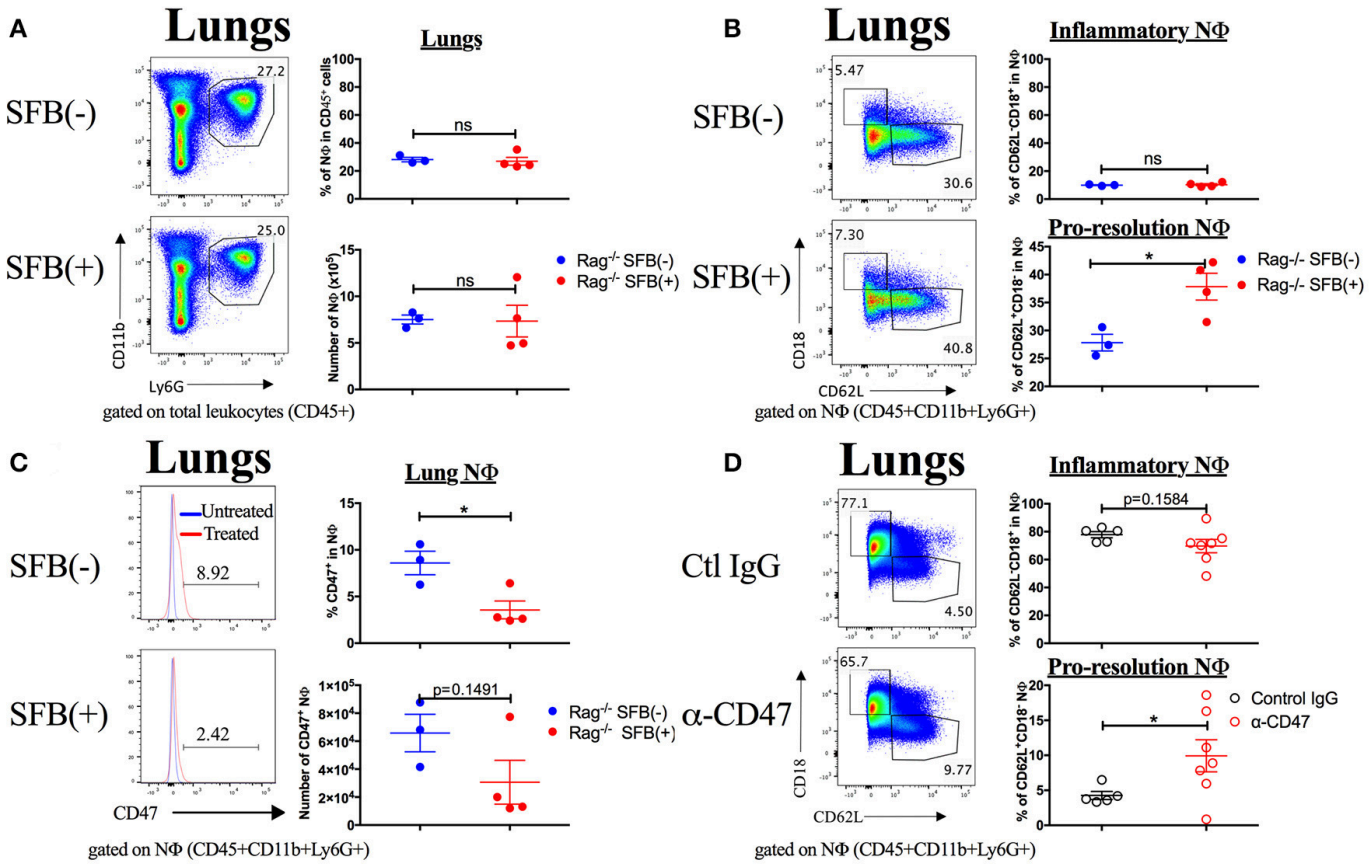
SFB alters lung neutrophil phenotype in Rag^−/−^ mice treated with heat-inactivated *S. pneumoniae*, and α-CD47 blocking antibodies alone alter neutrophil phenotype. **(A–C)** 10^8^ CFU equivalents of heat-inactivated *S. pneumoniae* were administered i.n. to SFB(−) and SFB(+) Rag^−/−^ mice at 2 time points, 12 h apart. Tissues were removed and analyzed 3 days after the initial treatment. **(A)** Representative plots and compiled graphs of frequency (as a percentage of total leukocytes) and number of lung neutrophils in heat inactivated *S. pneumoniae*-treated SFB(−) and SFB(+) Rag^−/−^ mice, as indicated. **(B)** Representative plots and compiled graphs of frequency (as a percentage of total neutrophils) of inflammatory or pro-resolution neutrophils in Rag^−/−^ lungs at 3 days post treatment. **(C)** Representative histograms and compiled graphs of frequency (as a percentage of total neutrophils) of lung neutrophils expressing CD47 in Rag^−/−^ mice at 3 days post treatment with heat-inactivated *S. pneumoniae*. (*N* = 3-4/group, 1 assay). **(D)** α-CD47 or control IgG was administered i.p. 1 day before i.n. infection with *S. pneumoniae*. Representative plots and compiled graphs of frequency (as a percentage of total neutrophils) of inflammatory or pro-resolution neutrophils in Rag^−/−^ lungs at 3 days post infection. (*N* = 5–7/group, 3 assays combined). Bars represent the means ± standard error. ^*^p < 0.05.

One potential additional mechanism that might contribute to SFB-mediated neutrophil phenotype changes during *S. pneumoniae* lung infection could be enhanced bacterial clearance by SFB colonization, leading to a reduced overall bacterial load. In this case, the larger changes in neutrophil phenotype from inflammatory to pro-resolution at day 3 post infection in SFB(+) compared to SFB(−) Rag^−/−^ mice would be secondary to the better bacterial clearance in SFB(+) mice. To address this, we administered heat-inactivated bacteria, at a dose two logs higher than our highest live bacteria infection, in two doses 12 h apart (Duong et al., 1998; Dominis-Kramaric et al., 2011). In this way, we can control for potential reduced bacterial load at day 3 post infection due to better clearance in SFB(+) compared to SFB(−) Rag^−/−^ mice, since each individual receives the same amount of bacterial antigen, without the potential for change during infection. When we analyzed the tissues 3 days after the initial treatment, we found that there was no change in frequency or number of neutrophils in the lungs (Figure 9A). However, the frequency and number of neutrophils in the lungs was much lower than that seen in a live bacterial infection, perhaps obscuring any potential decrease (Figure 9A, compare Figure 7A). Nevertheless, we did observe an enrichment of pro-resolution neutrophils in the lungs of SFB(+) Rag^−/−^ mice compared to their SFB(−) counterparts (Figure 9B, Supplementary Figure 6A). This suggests that the shift in neutrophil phenotype seen in *S. pneumoniae* infection in SFB(+) compared to SFB(−) Rag^−/−^ mice is not due solely to better control of bacterial expansion, although this experiment does not rule out better bacterial clearance as a potential additional mechanism. In addition, we observed a decrease in CD47 expression on lung neutrophils from SFB(+) Rag^−/−^ mice that received heat-inactivated *S. pneumoniae*, compared to the SFB(−) group (Figure 9C). These results support the conclusion that SFB is able to modulate CD47 levels in lung neutrophils independently of bacterial control. Altogether, these data suggest that SFB may benefit the host during *S. pneumoniae* lung infection through modulation of lung neutrophil phenotype and CD47 expression levels.

Finally, in order to determine whether direct CD47 modulation could cause the changes in neutrophil phenotype that we observed in SFB(+) Rag^−/−^ mice, we treated SFB(−) Rag^−/−^ mice with a CD47 antagonistic antibody (α-CD47), which has been shown to increase the rate of efferocytosis *in vivo* (Kojima et al., 2016). We hypothesized that this treatment would recapitulate the effects of SFB colonization on neutrophils during *S. pneumoniae* lung infection. Indeed, we found that α-CD47 treatment itself in SFB(−) Rag^−/−^ mice induced an increase in pro-resolution neutrophils and a trend toward a decrease in inflammatory neutrophils in comparison to control IgG-treated group (Figure 9D, Supplementary Figure 6B). The concordance of these results with our findings in SFB(+) Rag^−/−^ mice provides further evidence for modulation of CD47 as a mechanism by which SFB manipulates the lung neutrophil phenotypes.

## Discussion

By using a unique model involving *S. pneumoniae* infections in Rag^−/−^ mice lacking T and B cells, we were able to specifically study pathogenesis and immunoprotection in the lungs in immunodeficient Rag^−/−^ hosts and how the gut microbiota may impact these processes. This is a crucial area, as *S. pneumoniae* pulmonary infections in immunocompromised individuals have grown in recent years, and mortality remains high in these patients (Fernandez Guerrero et al., 2003; Quan et al., 2016). Many of these immunocompromised patients have a severe deficiency in T cells and/or B cells, such as in HIV patients and the elderly population (Aberle et al., 2013; Nasi et al., 2017). Notably, we elucidate a role for microbiota protection through the gut–lung axis in immunodeficient Rag^−/−^ mice by demonstrating that a gut commensal, SFB, may promote disease resolution of the inflammatory response post infection, which lead to an improvement of the overall outcome for the host. Specifically, our data indicate that in the deficiency of adaptive immunity, the gut commensal SFB may provide an additional, essential layer of protection for the host in the lungs, by promoting neutrophil resolution.

It has been demonstrated that colonization with specific commensal species such as SFB induces natural serum IgM and gut IgA production (Thurnheer et al., 2003), and our group has also shown that SFB increases the antibody response to an autoantigen through induction of T follicular helper (Tfh) cells (Teng et al., 2016). Here, we show for the first time that SFB can help boost systemic antibodies in a T cell-independent manner. Importantly, our data also demonstrate that B but not T cells are the critical immune component that provides protection against acute *S. pneumoniae* lung infection. Our data is consistent with results from other groups demonstrating that B cells form a critical part of the anti-*S. pneumoniae* immune response (Briles et al., 1981; Haas et al., 2005). B cell-mediated protection could potentially be effected by production of natural antibodies and TI-II antibodies recognizing *S. pneumoniae*, since the disease progresses acutely. For example, Briles et al showed that mice defective in production of natural and TI-II antibodies were protected by injection of IgG3 anti-phosphorylcholine antibodies, which react to phosphorylcholine expressed on *S. pneumoniae*, whereas control mice were highly susceptible to *S. pneumoniae* infection (Briles et al., 1981). Interestingly, although SFB colonization helps the host to produce TI-II antibodies or maintain natural antibodies that bind *S. pneumoniae*, we found that the basal level of B cell activity in the absence of SFB is sufficient to raise the host's resistance against severe *S. pneumoniae* infection, as B cell transfer alone can rescue Rag^−/−^ mice from the severe temperature drop that occurs in SFB(−) hosts. We and others have previously reported that SFB induces T helper 17 (Th17) cells (Ivanov et al., 2009; Bradley et al., 2017), and other studies found that SFB protects against acute *Staphylococcus aureus* pneumonia and *Aspergillus fumigatus* lung fungal infection by up-regulating Th17 responses (Gauguet et al., 2015; Mcaleer et al., 2016). In an acute infection, our results demonstrate that T cells are dispensable for host resistance against *S. pneumoniae* lung infection regardless of SFB status. This is likely due to the fact that 3 days of infection is too short a time to mount a Th17 response.

Clearance of apoptotic neutrophils by phagocytes (a.k.a. efferocytosis) enables maintenance of tissue homeostasis as well as the resolution of inflammation following infection or injury (Greenlee-Wacker, 2016). Our data indicate that in immunocompromised Rag^−/−^ mice, following a drastic rise in neutrophils 18 h post *S. pneumoniae* infection, by day 3 post infection, there was already a decline of neutrophils in the lung. Strikingly, our data indicate a novel observation that gut SFB colonization facilitates a sharper decline in lung neutrophils when compared to SFB(−) Rag^−/−^ mice. When examined closely, our results indicate that SFB-colonized hosts display a reduction in activated, inflammatory neutrophils (CD62L^−/lo^CD18^hi^). Campbell et al. found that increased CD62L was co-occurrent with decreased expression of inflammation-associated genes and increased expression of tissue remodeling and angiogenesis genes involved in wound healing and repair, suggesting an anti-inflammatory, pro-resolution phenotype (Campbell et al., 2016). Our results further demonstrate that coincident with the reduction in inflammatory lung neutrophils, there is also an induction of pro-resolution, CD62L^hi^ neutrophils in the lungs of SFB(+) hosts. Importantly, the pro-resolution phenotype in neutrophils still occurred more strongly in SFB(+) than SFB(−) Rag^−/−^ mice when treated with dead bacteria, indicating that the SFB-mediated neutrophil phenotype is not merely a secondary response to reduced bacterial load. It has been shown previously that SFB can protect against the intestinal pathogen *Enteramoeba histolytica* by modulating the innate compartment, particularly by increasing neutrophil frequency in the intestines (Burgess et al., 2014, 2016). In addition, microbiota-derived peptidoglycan in the serum primes neutrophils in the bone marrow through NOD1-dependent sensing, enhancing their ability to kill *S. pneumoniae* (Clarke et al., 2010). Our report adds to the body of literature on the gut microbiota's immunomodulatory effects, suggesting that the gut microbiota may regulate neutrophils at a gut-distal site in the lung.

During infection, neutrophils undergo active apoptosis and expose phosphatidylserine, which is one of the most symbolic “eat-me” signals leading to efferocytosis (Birge et al., 2016). CD47 delivers a “don't eat me” signal by interacting with SIRPα on phagocytes, leading to phosphorylation of the ITIM signaling regions on SIRPα's intracellular domain, and subsequent inhibition of engulfment (Barclay and Van den Berg, 2014). When CD47 is down-regulated or missing, pro-engulfment molecules, such as phosphatidylserine, interact with their receptors, leading to actin remodeling and cytoskeletal rearrangement in the macrophage to allow efferocytosis to take place (Gardai et al., 2005). Previously, *S. aureus* has been shown to up-regulate CD47 leading to inhibition of efferocytosis and adverse outcomes for the host. Similar to *S. aureus*, our data show that *S. pneumoniae* also may target efferocytosis by up-regulating CD47 expression on neutrophils, potentially promoting tissue damage. Importantly, our results demonstrate that SFB colonization in the gut is associated with down-regulation of CD47 specifically on inflammatory neutrophils in the lung, which may support better disease resolution compared to SFB(−) hosts, demonstrated by reduced bacterial burdens, lung pathology, and temperature loss in SFB(+) compared to SFB(−) Rag^−/−^ mice. In addition, we demonstrated that blocking CD47 alone in SFB(−) Rag^−/−^ mice resulted in effects on lung neutrophil phenotype comparable to those observed in SFB(+) Rag^−/−^ mice. Our data is supported by reports demonstrating efferocytosis to be a crucial part of controlling infection and promoting bacterial clearance (Capasso et al., 2016; Jorgensen et al., 2016). Furthermore, CD47 down-regulation leads to better resolution post-infection and it has been shown that deficiency in CD47 results in lower bacterial burdens and increased survival of mice after infection (Gresham et al., 2000). It should be noted that CD47 has also been implicated in migration of neutrophils and other leukocytes, although this is still a subject of debate; for example, one group demonstrated that anti-CD47 blocking antibodies inhibit neutrophil transmigration *in vitro* (Chin et al., 2009), while another group showed that CD47 deficient neutrophils were not impaired in migration, but that CD47 deficient mice were unable to replenish depleted neutrophils at the same rate as WT mice (Bian et al., 2013). Therefore, it is possible that SFB's effect on CD47 downregulation in inflammatory lung neutrophils may promote resolution through mechanisms additional to increasing efferocytosis susceptibility.

Because of the paucity of data regarding the microbiota's impact on neutrophil resolution, we focused our work on studying neutrophil resolution as one of the potential mechanisms for SFB-mediated resistance against *S. pneumoniae*. However, our observation that SFB colonization results in more stable temperatures throughout the course of acute *S. pneumoniae* lung infection, rather than solely in the resolution phase, suggests that SFB-mediated effects on neutrophil resolution may be preceded by other mechanisms involved in the response to *S. pneumoniae*. The reduction in lung inflammation in SFB(+) compared to SFB(−) Rag^−/−^ mice, as shown by the lung cytokine milieu, could be a result of better bacterial clearance and/or neutrophil resolution. Our data also show a trend toward a reduction in bacterial burden in SFB-colonized mice during the height of infection at 18 h post infection, which suggests that it is possible that SFB may contribute to enhanced bacterial clearance before the resolution phase at day 3 post infection. One potential mechanism for the enhanced bacterial clearance at 3 days post infection may be enhanced phagocytosis of bacteria. Indeed, the gut microbiota has been demonstrated previously to exert an impact on host leukocyte phagocytosis (Schuijt et al., 2016). Because we failed to observe an increase in lung neutrophil numbers during the height of infection at 18 h by SFB, it seems less likely that enhanced neutrophil recruitment would be driving SFB-mediated protection in our model, as has been suggested in other models (Anthony et al., 2013; Gauguet et al., 2015). However, it is possible that SFB could promote functionality of innate immune responders in the lungs, either by directly enhancing phagocytic abilities, or by enhancing their localization to the most affected areas (Gray et al., 2017). Clearly, further investigation into this model is needed to further elucidate the mechanisms by which the gut commensal SFB enhances resistance to *S. pneumoniae* lung infection.

In conclusion, by selectively studying *S. pneumoniae* infection using immunodeficient Rag^−/−^ mice, we found that gut commensals may provide additional protection for the adaptive-immunodeficient host by regulating host innate immune responses. Importantly, control of neutrophilic inflammation at the resolution phase is known to increase host survival against *S. pneumoniae* (Bhowmick et al., 2013; Bou Ghanem et al., 2015). Specifically, depletion of neutrophils at the beginning of an *S. pneumoniae* infection decreased host survival, while neutrophil depletion 18 h post infection significantly improved survival. Supported by these previous reports, our data suggest that gut commensal bacteria can modulate the neutrophil response in the lungs and may promote host resistance by modifying neutrophil phenotype through down-regulating neutrophil expression of an efferocytosis-inhibitory molecule. However, these data cannot rule out that additional mechanisms, such as enhanced clearance of bacteria, may also contribute to the improved outcomes seen in SFB(+) Rag^−/−^ mice. Importantly, these results have implications for human disease, suggesting that colonization with certain immunomodulatory commensals may be beneficial for immunocompromised individuals to reduce susceptibility to severe pneumonia due to uncontrolled inflammation. Pro-resolving drugs such as glucocorticoids have been used in clinical trials, with mixed results (Wirz et al., 2016). However, it is possible that continuous low-level signaling by the microbiota might have a more beneficial effect on neutrophil resolution compared to large doses of glucocorticoids administered after infection has been diagnosed. Additional studies on commensal-mediated protection against *S. pneumoniae* infection are crucial, as they may help pave the way for future treatment of pneumococcal pneumonia.

## Ethics statement

This study was carried out in accordance with the recommendations of the Institutional Animal Care and Use Committee at the University of Arizona under the protocol reference number 11-278.

## Author contributions

KF and H-JW conceived and designed the study and wrote the manuscript. KF and CK designed and performed the experiments and analyzed the data. IJ, HM, and WR performed *S. pneumoniae* CFU analysis, SFB quantification and ELISA analysis. T-VN and KD contributed to the design of the lung histology experiments and performed H&E staining and analysis of lung histology.

### Conflict of interest statement

The authors declare that the research was conducted in the absence of any commercial or financial relationships that could be construed as a potential conflict of interest.
