# Supplementary figures and images for: Gut Microbiota Contributes to Resistance Against Pneumococcal Pneumonia in Immunodeficient Rag−/− Mice
Source: Front Cell Infect Microbiol. 2018 Apr 18;8:118. doi: 10.3389/fcimb.2018.00118 (PMC5932343; doi:10.3389/fcimb.2018.00118)

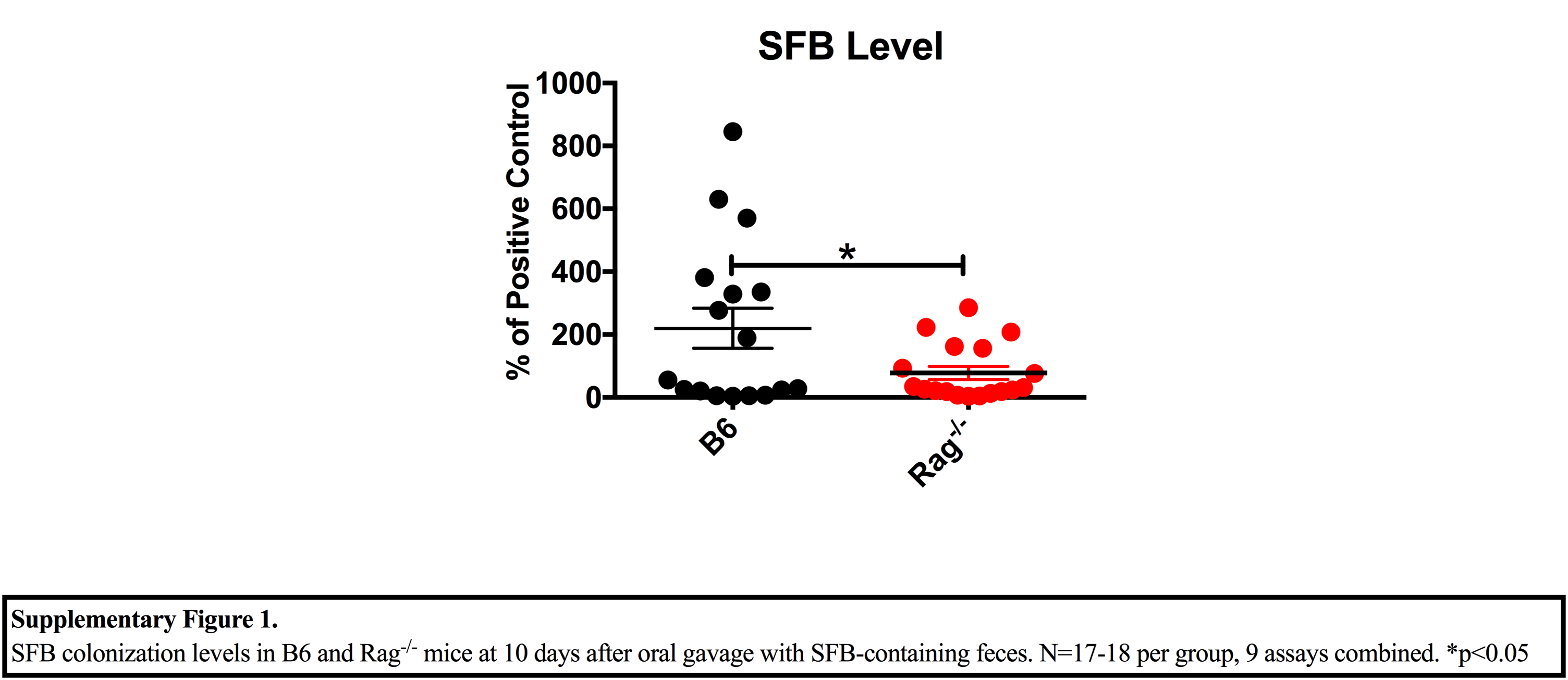

Supplement: Supplementary file 1 [file Image1.TIFF]

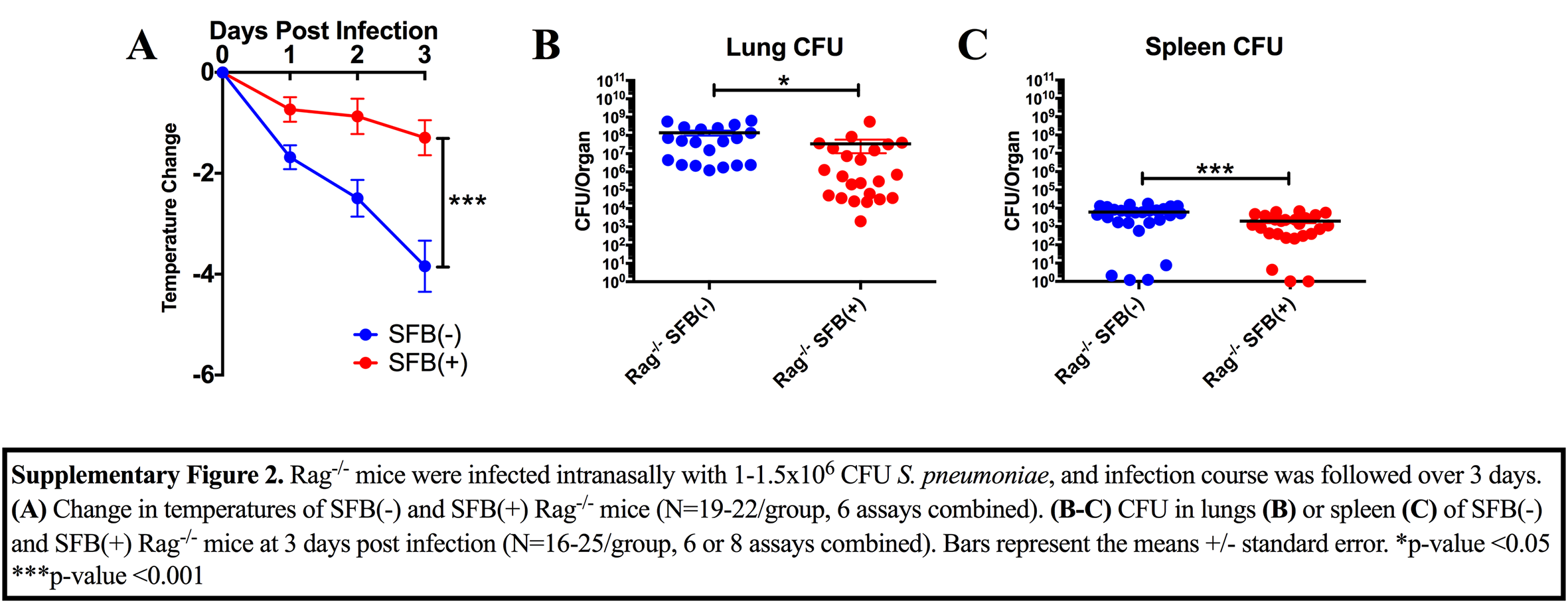

Supplement: Supplementary file 2 [file Image2.TIFF]

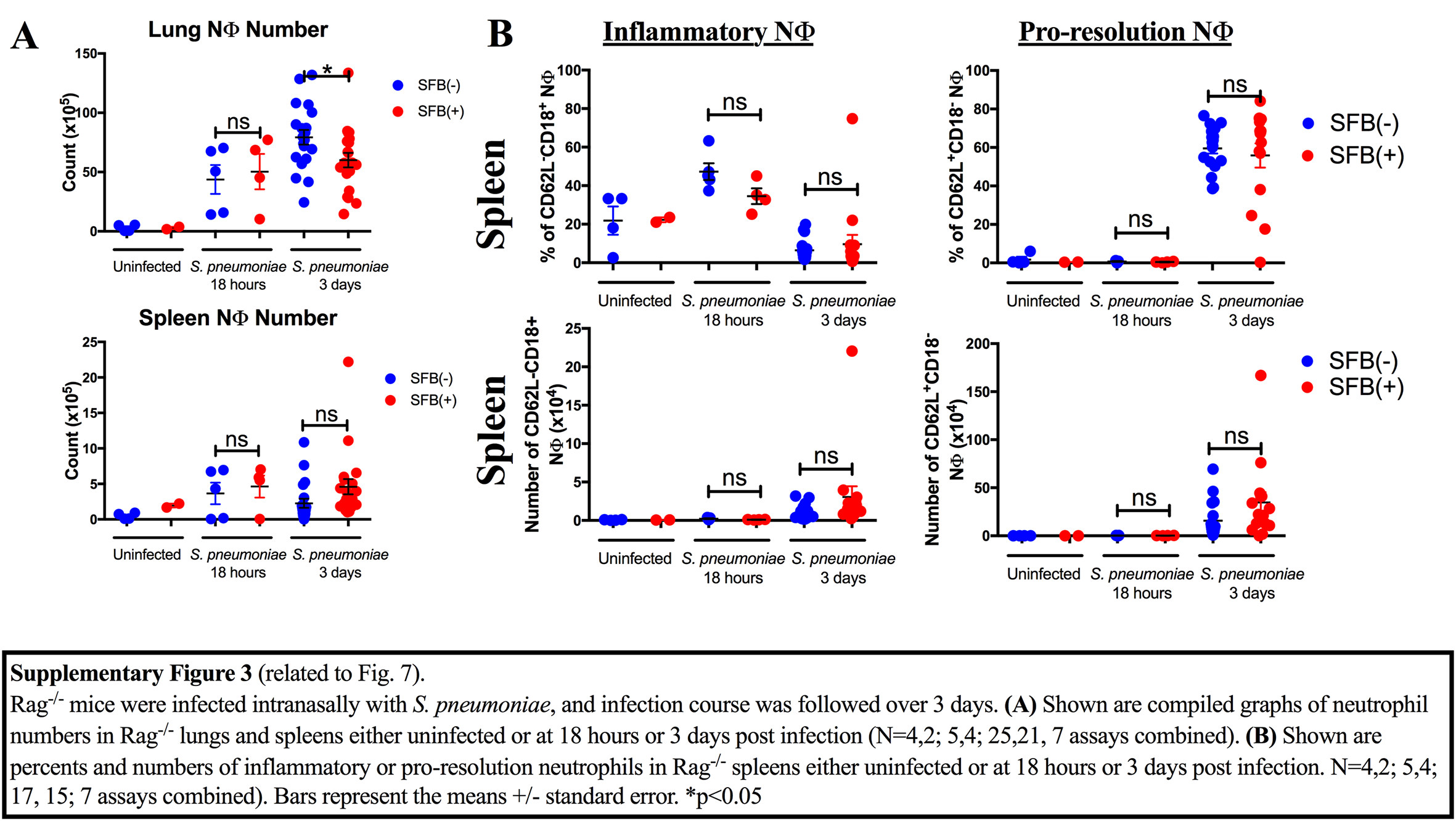

Supplement: Supplementary file 3 [file Image3.tiff]

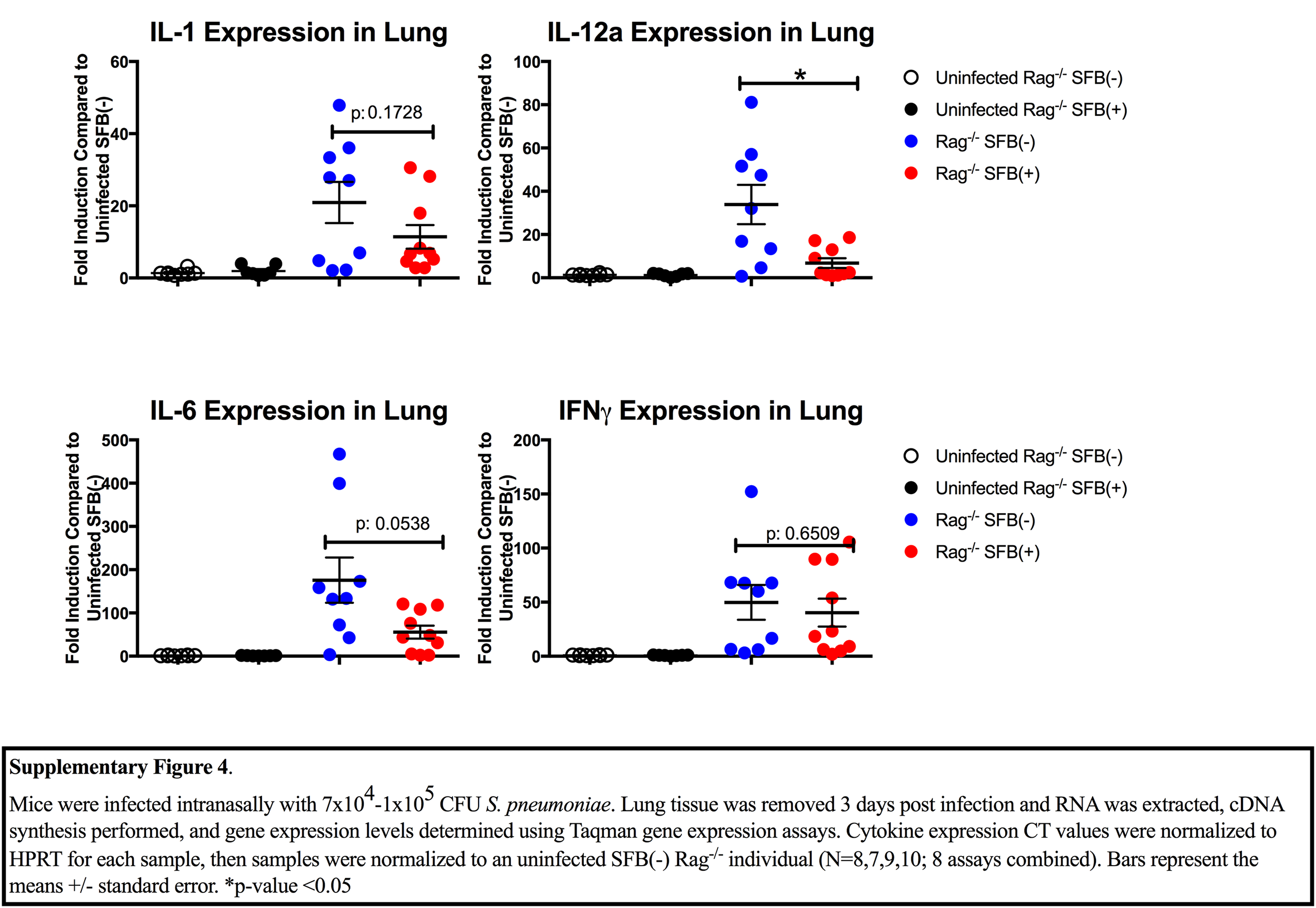

Supplement: Supplementary file 4 [file Image4.TIFF]

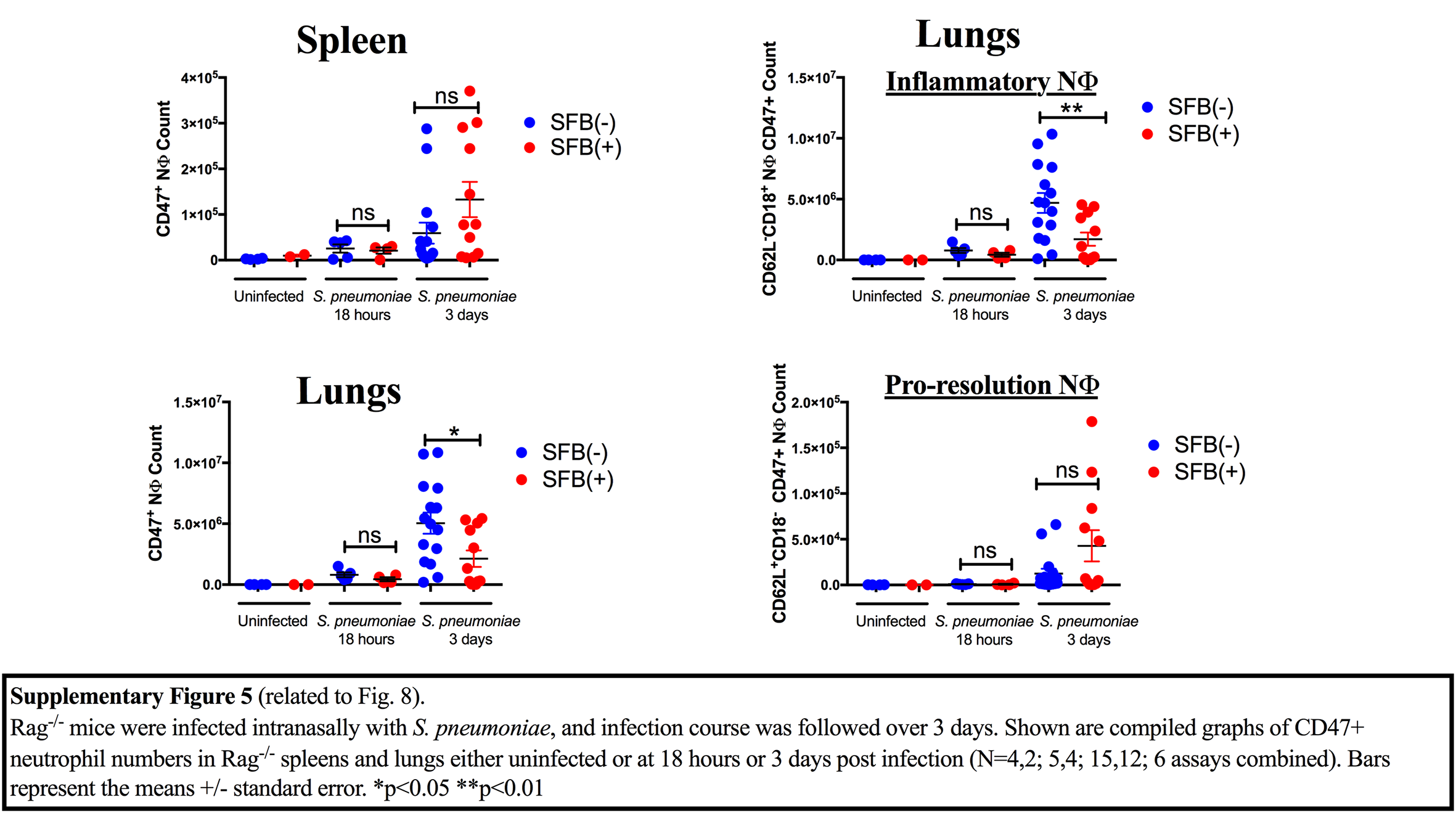

Supplement: Supplementary file 5 [file Image5.tiff]

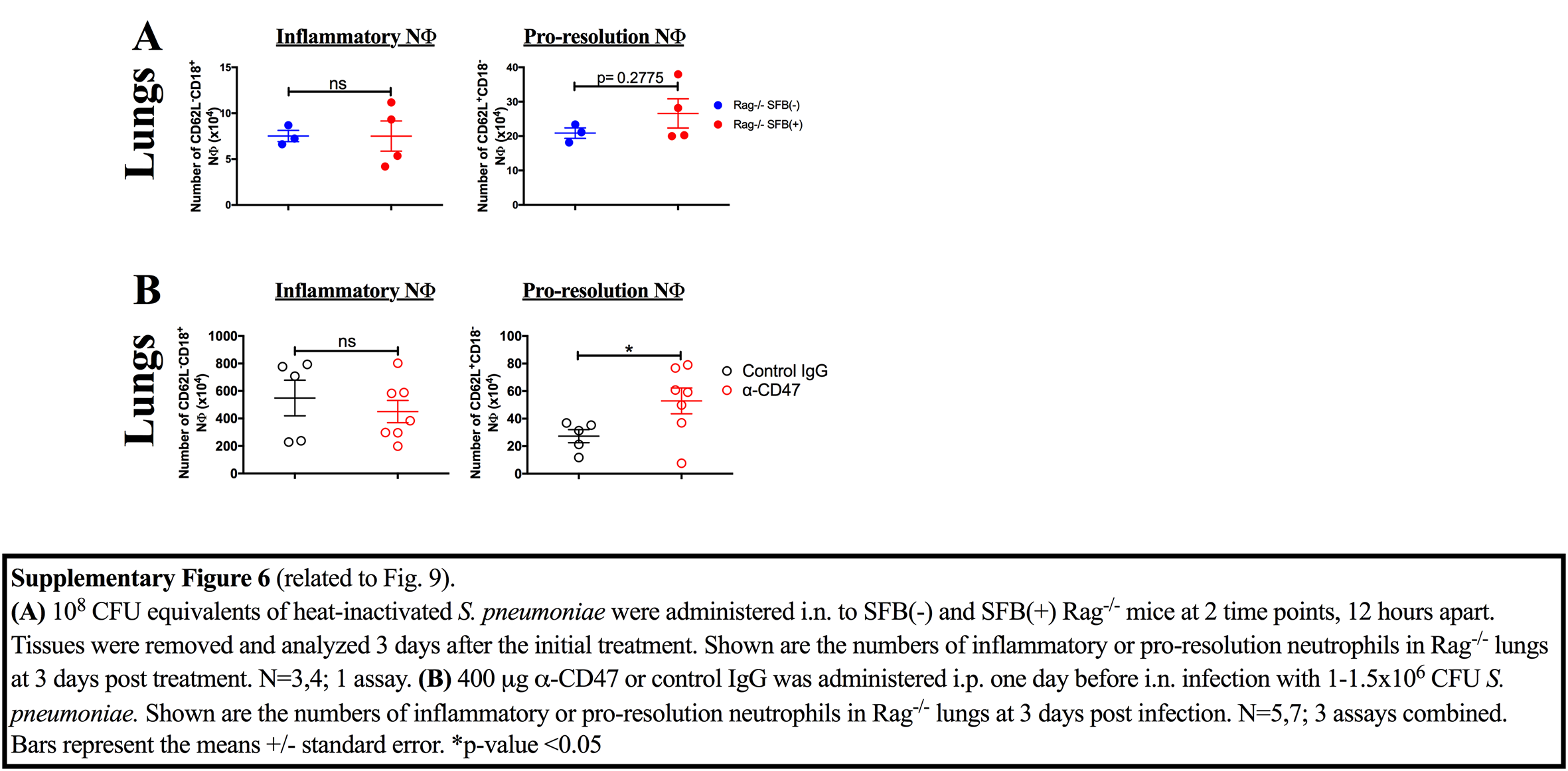

Supplement: Supplementary file 6 [file Image6.tiff]
